# Integrating Molecular Biomarker Inputs Into Development and Use of Clinical Cancer Therapeutics

**DOI:** 10.3389/fphar.2021.747194

**Published:** 2021-10-19

**Authors:** Anna D. Louie, Kelsey Huntington, Lindsey Carlsen, Lanlan Zhou, Wafik S. El-Deiry

**Affiliations:** ^1^ Laboratory of Translational Oncology and Experimental Cancer Therapeutics, Warren Alpert Medical School, Brown University, Providence, RI, United States; ^2^ Department of Surgery, Lifespan Health System and Brown University, Providence, RI, United States; ^3^ Pathobiology Graduate Program, Warren Alpert Medical School, Brown University, Providence, RI, United States; ^4^ Department of Pathology and Laboratory Medicine, Warren Alpert Medical School, Brown University, Providence, RI, United States; ^5^ Joint Program in Cancer Biology, Lifespan Health System and Brown University, Providence, RI, United States; ^6^ Cancer Center at Brown University, Providence, RI, United States; ^7^ Hematology/Oncology Division, Department of Medicine, Lifespan Health System and Brown University, Providence, RI, United States

**Keywords:** biomarkers, cancer therapeutics, genomics, machine learning, liquid biopsy

## Abstract

Biomarkers can contribute to clinical cancer therapeutics at multiple points along the patient’s diagnostic and treatment course. Diagnostic biomarkers can screen or classify patients, while prognostic biomarkers predict their survival. Biomarkers can also predict treatment efficacy or toxicity and are increasingly important in development of novel cancer therapeutics. Strategies for biomarker identification have involved large-scale genomic and proteomic analyses. Pathway-specific biomarkers are already in use to assess the potential efficacy of immunotherapy and targeted cancer therapies. Judicious application of machine learning techniques can identify disease-relevant features from large data sets and improve predictive models. The future of biomarkers likely involves increasing utilization of liquid biopsy and multiple samplings to better understand tumor heterogeneity and identify drug resistance.

## Introduction

**FIGURE 1:**
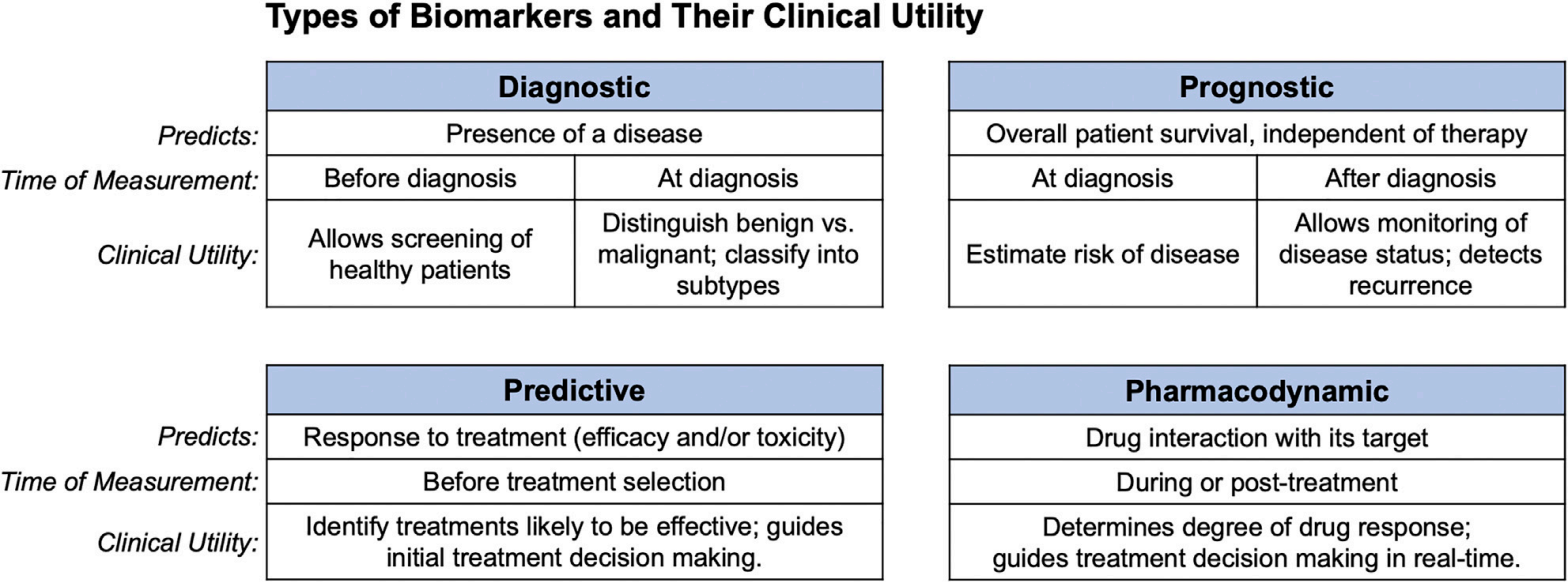
Clinical uses of biomarkers. Diagnostic, prognostic, predictive, and pharmacodynamic biomarkers are shown along with what each predicts, and the clinical setting in which they can be used.

A biomarker is a measurable indicator that predicts disease presence, severity, or response to treatment. Levels of biomarkers can be clinically useful by guiding disease diagnosis, or by revealing the pharmacodynamics of drug treatment. Figure 1 depicts various types of biomarkers and their potential for clinical utility.

**FIGURE 2:**
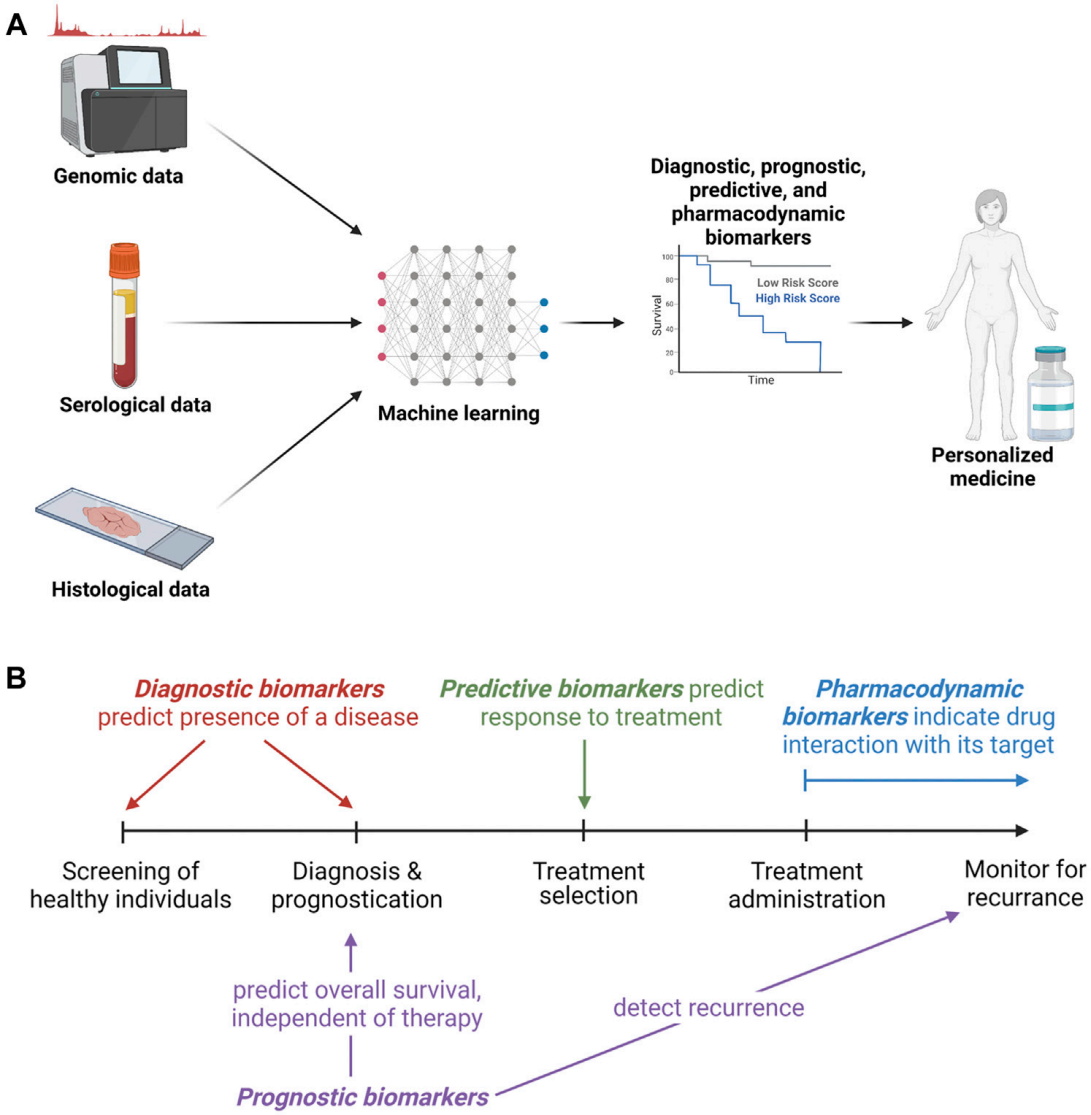
Biomarker development and clinical utility. **(A)**. Overview of methods of biomarker development, testing and clinical utilization. **(B)**. Types of biomarkers with a timeline of opportunities for utilization.

Approved and experimental biomarkers can be classified based on their clinical uses. These clinical uses parallel the progressive utilization of biomarkers during the development of cancer therapeutics. Figure 2 gives an overview of biomarker development strategies and potential uses. Biomarkers are divided into categories including diagnostic, prognostic, pharmacodynamic, and predictive, with some falling into several categories. This review briefly summarizes some current clinical uses of biomarkers and their effect on development and application of cancer therapeutics. It also addresses promising strategies for biomarker discovery such as genomics, proteomics and machine learning, and discusses the increased clinical accessibility and potential applications of liquid biopsies.

### Companion Diagnostics

Companion diagnostics is the development of predictive biomarkers in conjunction with novel therapeutics. It identifies patients who are likely to respond to the treatment or to experience severe toxicity. An early example is estrogen receptor assays which are implemented in the prescription of the estrogen receptor modulator tamoxifen. Since then, others have been developed including measurement of HER2 levels prior to treatment of breast cancer with the anti-HER2 antibody pertuzumab, and measurement of PD-L1 levels prior to treatment with the anti-PD-L1 antibody pembrolizumab (Jørgensen et al., 2016). Companion diagnostics increasingly subdivide patients based on molecular biomarkers, which may be required to direct prescription of targeted therapies. This codependence is reflected in FDA approvals of companion biomarkers in conjunction with novel therapeutics, such as the simultaneous approval of vemurafenib and an assay to detect the V600E mutation it targets (Scheerens et al., 2017). Companion diagnostics allow improved patient selection for drug trials and quicker identification of clinically effective drugs for personalized treatments.

### Diagnostic Biomarkers

While companion diagnostics focuses on predictive biomarkers, all types are utilized in both patient care and the phases of drug development. Diagnostic biomarkers suggest the presence of a disease or can classify patients into subtypes. Elevated levels of these diagnostic biomarkers may suggest the presence of cancer, and thus can be used as a screening tool in healthy individuals or can support other diagnostic measures such as imaging and biopsy. Several long-used cancer diagnostic biomarkers include prostate-specific antigen (PSA), used for diagnosis of prostate cancer (Welch and Albertsen 2009); cancer antigen 19-9 (CA 19-9), the gold standard serum biomarker for diagnosis of pancreatic ductal adenocarcinoma (PDAC) (Poruk et al., 2013); and CA 125, a classical biomarker in ovarian cancer (Felder et al., 2014). Evidence supporting the utility of cytokines as diagnostic biomarkers is evolving, including data demonstrating IL-6 and VEGF as possible diagnostic biomarkers in ovarian and gastric cancer (Monastero and Srinivas, 2017). Further validation of these cytokines is needed to uncover their diagnostic utility, either as independent biomarkers or in conjunction with classical biomarkers to increase sensitivity and specificity. While diagnostic biomarkers are often used for subtyping a known malignancy, such as in leukemia (Jiang et al., 2016), many lack the specificity needed for cancer diagnosis in the general population (Califf 2018).

### Prognostic Biomarkers

Prognostic biomarkers predict the patient’s overall survival, independent of therapy. Examples of diagnostic biomarkers with prognostic value include CA 19-9 and CA 125, which can predict overall survival in PDAC and ovarian cancer, respectively (Poruk et al., 2013; Felder et al., 2014). Carcinoembryonic antigen (CEA) indicates poor overall survival in colorectal, breast, and lung cancer patients, though it is only regularly used for prognostication in colorectal cancer (CRC) (Dixon et al., 2003). Other types of biomarkers can also have prognostic value, such as miRNA-155 in hepatocellular carcinoma, which increases Wnt signaling pathway activity and is suggestive of a poor clinical prognosis (Nalejska et al., 2014). Even the presence of circulating tumor cells is correlated with metastasis and can serve as a marker of poor prognosis in non-metastatic breast cancer (Lucci et al., 2012). The prognostic information of biomarkers can guide treatment decision-making, monitor disease progression, and detect recurrence.

### Pharmacodynamic Biomarkers

Pharmacodynamic biomarkers suggest whether a drug has reached its target and exerted a cellular response (Jackson 2012). For example, measurement of Mitogen-Activated Protein Kinase (MAPK) pathway inhibition (*via* measurement of pERK) in non-small-cell lung cancer (NSCLC) patients receiving BRAF inhibitors can indicate direct drug-target interaction (Gainor et al., 2014). Such pathway-specific measurements can be taken simultaneously with markers of tumor cell proliferation (cyclin D1, Ki67) or tumor growth [*via* fludeoxyglucose (18F) measured by PET/CT] to determine first if the drug is hitting its primary target and second if the drug is mediating tumor suppression (Kelloff et al., 2005; Gainor et al., 2014). These measurements can determine the degree of response to the drug in clinical trials and guide treatment decision making in real-time. Most pharmacodynamic biomarkers are measured with tumor biopsies, but recently there has been increasing interest in less invasive blood-based biomarker development (Jackson 2012). Further study of pharmacodynamic biomarkers could personalize treatment doses for patients and provide a method to both minimize toxicity and avoid subtherapeutic dosing.

### Predictive Biomarkers and An Example of Biomarker Application

Predictive biomarkers indicate how patients are likely to respond to treatment, either in terms of efficacy or toxicity (Alves et al., 2019). They can be measured before first-line treatment or to choose a salvage therapy. Well-established predictive biomarkers include HER2 overexpression which predicts breast cancer response to anti-HER2 therapies like trastuzumab and KRAS, NRAS and BRAF mutations which predict resistance to Epithelial Growth Factor Receptor (EGFR) inhibitors in CRC (Jørgensen et al., 2016). Using EGFR therapy in CRC may lead to shorter survival in patients with certain mutations in these MAPK pathway genes, making them biomarkers of resistance to cetuximab (Boussios et al., 2019). More recent developments indicate that high circulating levels of IFN-γ predict response to immunotherapies such as immune checkpoint blockade (Karachaliou et al., 2018). Other predictive biomarkers forecast pharmacodynamic resistance or toxicity. Examples include genetic alterations in dihydropyrimidine dehydrogenase (DPD) and of UDP glucuronosyltransferase family one member A1 (UGT1A1), the enzymes responsible for inactivation of 5-FU and irinotecan, respectively. Genetic alterations that reduce the activity of these enzymes result in severe toxicity after treatment with the compound. Additionally, enhanced expression of excision repair cross-complementation group 1 (ERCC1) enhances DNA excision repair and leads to resistance to platinum-based drugs (Chung 2021). These predictive biomarkers guide initial treatment decisions by identifying potentially successful drugs and minimizing toxicity.

The clinical application of disease-related biomarkers can parallel their integration into drug development. For example, the use of biomarkers in breast cancer evolved to include diagnostic, prognostic, pharmacodynamic, and predictive biomarkers as the treatments and understanding of the disease progressed. Diagnostic biomarkers such as hormone receptor (HR) status are used to differentiate molecular subtypes of breast cancer. HR status was found to be associated with survival, making it also a prognostic biomarker (American Cancer Society Inc, 2019). Prognostic markers such as hormonal status, HER2 expression, and the 21-gene expression assay Oncotype DX have all been integrated into care and treatment decisions for breast cancer patients. Oncotype DX can predict chances of recurrence and this prediction is used clinically to evaluate the risks and benefits of adjuvant chemotherapy in patients with early stage HR positive breast cancer (Wang et al., 2019). The American Society of Clinical Oncology recommends the use of Oncotype Dx to guide the use of chemotherapy after surgery for patients with HR positive, HER2 negative early stage breast cancer, showing integration of multiple gene and protein expression biomarkers into clinical best practice recommendations for selection of therapeutics (Andre et al., 2019). As estrogen receptor modulators and aromatase inhibitors became available, HR status was also used as a predictive biomarker for endocrine therapy (Duffy et al., 2017). Ki67 is a marker of cell proliferation, and a pharmacodynamic response in Ki67 expression after treatment with endocrine therapy is an indication of on-target drug effects (Freelander et al., 2021). Mutations in the ESR1 gene encoding the estrogen receptor serve as a predictive biomarker of resistance to endocrine therapy (Dustin et al., 2019). This example shows how the development and clinical application of diagnostic, prognostic, pharmacodynamic, and predictive biomarkers can all be important in the development and application of targeted therapeutics.

## Genomics, Proteomics, and Machine Learning

Cancer-specific mutations are an appealing source of potential biomarkers. Genomic analyses have been applied to biomarker identification in both inherited and sporadic cancers. Early applications of gene mutations involved the identification of germline mutations that serve as prognostic biomarkers of elevated cancer risk, such as p53 mutations in Li-Fraumeni syndrome or BRCA mutations in hereditary breast cancer (Olivier et al., 2019). These tests expanded the role of genetic counselors and still guide screening and treatment recommendations (Weil, 2002). Other -omics applications have sought to further characterize tumor cells. Transcriptomics, epigenomics, metabolomics, and proteomics can all contribute information on tumor state. While integrated multi-omic approaches have not yet been fully integrated into therapeutic decision-making, new drug trials may incorporate this information into patient selection. For example, in PDAC, an immune profile combining whole-exome sequencing, RNA transcriptomics, and cell-surface protein expression has been developed to identify patients who are more likely to respond to immunotherapy (Lenzo et al., 2021).

### Genomics

The convoluted mutational profile of most sporadic cancers increases the complexity of genomic analysis. Advances in sequencing technologies including single-cell sequencing have further underscored tumor heterogeneity and provided insight into the variation in patient responses to therapy. Predictive biomarkers when combined with targeted therapies have shifted treatment decisions from a focus on tumor type to gene-directed individualized treatment plans. For instance, FDA-approved biomarkers such as activating mutations in EGFR predict effectiveness of EGFR inhibitors like gefitinib (Tsimberidou et al., 2020). Patients selected by EGFR mutation biomarker for gefitinib treatment have a 65% response rate, compared to 20–30% in unselected patients (Feng et al., 2021). Improvements in the accuracy and standardization of sequencing and reporting have increased the clinical utilization of large multi-gene panels and whole genome analysis.

### Proteomics

Proteomics attempts to directly analyze the main mediators of cellular function by quantifying protein activity and location (Olivier et al., 2019). The dynamic nature of the proteome may allow for better biomarkers of response to treatment and cancer surveillance. Proteomics can clarify the role of the tumor microenvironment (TME). Cell surface urokinase plasminogen activator receptor is an example of a prognostic biomarker that emerged from proteomic analyses. Mass spectrometry methods have also been FDA approved for analysis of the human microbiome, and are being assessed for relevance to colorectal and lung cancers (Su et al., 2021).

### Machine Learning

Many genomic and proteomic datasets are large, nonlinear, and multidimensional. The high number of variables measured for each clinical sample can require sophisticated data analysis strategies to differentiate signal from noise and adjust for multiple comparisons. Machine learning can be used to make predictions that incorporate and simplify multivariate information and can determine which variables (e.g., genetic mutations) are relevant biomarkers. The methods to select disease-relevant features while eliminating redundancy and noise range from linear models to neural networks (Feng et al., 2021). In selecting relevant features, machine learning models can work solely from information within the annotated data set or can incorporate known biologic relationships such as in gene set variation analysis.

There are many existing web servers and bioinformatic analysis tools. Among pan-cancer human—omics datasets, web servers aggregating patient data are available for DNA mutation, methylation, mRNA, micro-RNA, long non-coding RNA, and protein information. DNA mutation servers include cBioPortal (its 102,589 samples include The Cancer Genome Atlas data), GSCALite and CaPSSA. DNA methylation is available at MEXPRESS, GSCALite, and MethSurv. There are many databases of mRNA data including GENT2, PROGgeneV2, LOGpc, SurvExpress, PRECOG, and Oncomine which all have more than 15,000 patient samples. OncoLnc combines mRNA micro-RNA and long-noncoding RNA data. Proteomics patient datasets are available through CPTAC, TCPAv3.0, and TRGAted (Zheng et al., 2020).

Model development typically involves separate training and testing data. The quality of a model is determined by how much more effectively it classifies test data than would be expected by other available means. Validation can be achieved through independent datasets, but ideally also involves animal experiments or clinical trials (Deo, 2015). Clinical applications of machine learning analyses include the Oncotype Dx scoring in breast cancer (Wang et al., 2019), and clinical trials of personalized combination therapies chosen based on predicted response (Boichard et al., 2020). The application of machine learning can identify novel biomarkers from relationships not readily apparent within large data sets and will be increasingly important in new multi-omic approaches.

## Liquid Biopsy

Biomarkers can be derived from tumor tissue, blood, other biologic fluids, and even imaging. Blood-based biomarkers, or liquid biopsies, have become increasingly attractive in patient care (Michela 2021). Liquid biopsies have advantages over traditional solid biopsies as they are non-invasive, cost-effective, and expedite time to diagnosis. The most studied cancer biomarkers in plasma or serum samples are circulating tumor cells (CTCs), circulating tumor DNA (ctDNA), and exosomes.

### Circulating Tumor Cells

CTCs are a rare, migratory cell population shed from a tumor and believed to play a role in metastasis. CTCs have both diagnostic and prognostic value across several tumor types including PDAC (Ankeny et al., 2016b), breast cancer (Cristofanilli et al., 2004), ovarian cancer (Poveda et al., 2011), colon cancer (Romiti et al., 2014), and metastatic castration-resistant prostate cancer (CRPC) (Bono et al., 2008). In PDAC, CTCs can be a biomarker at diagnosis and a marker of disease progression, though they are not yet part of general clinical practice (Ankeny et al., 2016a). In breast cancer, the number of CTCs before treatment and at the first follow-up visit are independent predictors of progression free survival (PFS) and overall survival (OS) (Cristofanilli et al., 2004). Moreover, in ovarian and colorectal cancers, elevated CTC numbers are correlated with a higher risk of progression and worse OS (Poveda et al., 2011; Romiti et al., 2014). Finally, in CRPC, CTC counts were more predictive of OS than the classical biomarker PSA (Bono et al., 2008).

### Circulating Tumor DNA

ctDNA is tumor-released single- or double-stranded DNA that enters the bloodstream and can be detected for diagnosis, guidance of treatment, and monitoring of disease progression. Recently, ctDNA has been used to guide clinical decision-making in several tumor types including CRC (Chen et al., 2021), NSCLC (Song et al., 2020), and metastatic breast cancer (MBC) (Darrigues et al., 2021). In CRC, postoperative serial ctDNA detection identified recurrence before radiological imaging and was predictive of high relapse risk (Chen et al., 2021). ctDNA has suggested a novel therapeutic rechallenge strategy for CRC based on evidence that resistance mechanisms to anti-EGFR therapy extinguish over time off of that therapy (Misale et al., 2014; Parseghian et al., 2019; Sartore-Bianchi et al., 2021). Moreover, in a prospective real-world study of NSCLC, ctDNA clearance during treatment was correlated with better OS (Song et al., 2020). The FDA has approved the use of liquid biopsy for analysis of sensitizing and resistance mutations in NSCLC due to multiple studies where the application of biomarkers derived from liquid biopsy successfully guided treatment decisions (Saarenheimo et al., 2019). In MBC, ctDNA was a prognostic factor of PFS and efficacy of treatment was effectively monitored by serial ctDNA analyses before radiological evaluation (Darrigues et al., 2021). Analysis of ctDNA has been employed to differentiate between the clinical scenarios of pseudoprogression and hyperprogression following treatment of patients with immune checkpoint blockade therapy (Jia et al., 2019; Ma et al., 2019). Pseudoprogression is transient enlargement of lesions followed by partial response, and hyperprogression is unexpectedly rapid disease progression during treatment (Frelaut et al., 2020). Distinguishing between pseudoprogression and hyperprogression determines whether patients should remain on therapy or discontinue therapy. The speed of ctDNA analyses is ideal as tumors accelerate their rate of growth during hyperprogression.

### Exosomes

Exosomes are small cell-derived vesicles that are shed from myriad cell types into biological fluids under normal and pathological conditions. They carry molecular constituents of their host cells, including proteins, lipids, mRNAs, and miRNAS (Zhou et al., 2020). They have been implicated in tumor development and metastasis, making them potential diagnostic biomarkers for several tumor types including gastrointestinal, breast, and lung cancers. In a gastrointestinal meta-analysis, a change in exosome expression was significantly correlated with poor OS (Zhang et al., 2021). Furthermore, circulating exosomal miRNAs were indicative of breast cancer (Hannafon et al., 2016) while exosomal proteins were indicative of lung cancer (Sandfeld-Paulsen et al., 2016).

## Biomarkers of Immunotherapy

Biomarkers are particularly important in immunotherapy as immune checkpoint inhibitors have demonstrated impressive responses across multiple tumor types. However, most patients do not benefit from this therapy and there is a growing need for biomarkers that can predict patient response.

### Tumor and Immune Cell Phenotype

Some patients with PD-L1 positive tumors have improved clinical outcomes. However, the utility of this biomarker is inconsistent across tumor types (Doroshow et al., 2021). Increased circulating levels of sPD-L1 have also been correlated with poor response to immunotherapy, and may be more predictive than tumor cell expression of PD-L1 in soft tissue sarcomas (Asanuma et al., 2020). Diversity of immune cell repertoires could also function as a biomarker of response, as effective T cell responses require a diversity in T-cell receptor (TCR) clonality. In NSCLC, patients with increased CD8+ TCR clonality after immunotherapy had improved PFS compared to those with decreased clonality (Han et al., 2020).

### Tumor Microenvironment

Immune status of the TME, including tumor-infiltrating immune cells and cytokine profiles, can be predictive biomarkers for immunotherapy. In melanoma, tumor-infiltrating immune cell subsets such as CD4+, CD8+, and FOXP3+ T cells correlate with improved treatment efficacy and disease outcome (Balatoni et al., 2018). An increased presence of cytotoxic T cells is generally predictive of clinical benefit from immunotherapy (Wei et al., 2021). In contrast, immunodepleted or immunodeficient tumors are less likely to respond. Furthermore, immunostimulatory TMEs characterized by inflamed IFN-γ profiles are predicted to respond better to immunotherapy (Gibney et al., 2016).

### Tumor Genomic Biomarkers

Genomic biomarkers such as tumor mutational burden (TMB), neoantigen load, and microsatellite status are all clinically relevant biomarkers of immunotherapy. Tumors with high TMB are thought to have increased neoantigen burden making them immunogenic and more responsive to immunotherapy. High TMB is correlated with response to immunotherapy in several cancer types including NSCLC (Hellmann et al., 2018b), melanoma (Goodman et al., 2017), and CRC (Schrock et al., 2019). Similarly, microsatellite status is significantly associated with response to immunotherapy, where mismatch repair deficient or microsatellite instability high tumors are associated with durable responses to immunotherapy and improved prognosis. This correlation has been shown in cancers like NSCLC (Hellmann et al., 2018a), melanoma (Kubecek et al., 2016), and CRC (Andr et al., 2020).

## Discussion and Conclusion

As the use of biomarkers in clinical cancer therapeutics advances, more frequent screening to evaluate drug efficacy and the development of resistance will be desirable. For patients undergoing these repeated screenings, the noninvasive nature of liquid biopsy is advantageous. ctDNA has been applied in detecting resistance to EGFR tyrosine kinase inhibitors and can replace a tumor biopsy in the decision to transition to third-generation EGFR tyrosine kinase inhibitors (Kilgour et al., 2020). While sequential targeting of the predominant mutation can prolong treatment responses, intratumoral heterogeneity can be a source of persistent residual disease and eventual drug resistance and recurrence. Multiregional sequencing, single-cell methods and sequential liquid biopsies allow more detailed evaluation of tumor heterogeneity with the potential to contribute multiple sub-population biomarkers and guide combinatorial treatment strategies (Lim and Ma 2019).

The complexities of tumor biology, its evolution over time and heterogeneity both between patients, and within a single tumor all add challenge to the development of biomarkers that meaningfully impact clinical cancer therapeutics. Biomarkers have the most clinical relevance when they are reproducibly and accurately measurable, clinically feasible, and prospectively validated in randomized clinical trials. Biomarkers are increasingly available at all phases of patient care, from screening and prevention to evaluations of drug efficacy and tumor response. Some biomarkers, such as those related to the EGFR pathway have already become approved decision-making tools for selecting cancer therapeutics. More are under development and may add insights to important areas of research such as tumor immune modulators and the tumor microenvironment. Blood-based biomarkers promise to reduce the potential for biopsy complications and make it easier to test repeatedly for tumor evolution. Many new trials are utilizing companion diagnostics where biomarker assays guide the use of targeted cancer drugs (Jørgensen et al., 2016). As the types of biomarkers expand, the information contained in published datasets also grows. Cross-validation among the increasing number of existing datasets will increase the power of machine learning algorithms and improve their clinical predictions (Shukla et al., 2015). While there are still many treatment decisions made without the aid of biomarkers, the future may yield more tools to guide the development and utilization of clinical cancer therapeutics.
